# Clinicopathological significance of endoplasmic reticulum stress proteins in ovarian carcinoma

**DOI:** 10.1038/s41598-020-59116-x

**Published:** 2020-02-07

**Authors:** Soma Samanta, Shuzo Tamura, Louis Dubeau, Paulette Mhawech-Fauceglia, Yohei Miyagi, Hisamori Kato, Rich Lieberman, Ronald J. Buckanovich, Yvonne G. Lin, Nouri Neamati

**Affiliations:** 1 Department of Medicinal Chemistry, College of Pharmacy, Rogel Cancer Center, University of Michigan, 1600 Huron Parkway, Ann Arbor, MI 48109 USA; 2 USC/Norris Comprehensive Cancer Center and Department of Pathology, Keck School of Medicine of USC, 1441 Eastlake Avenue, Los Angeles, CA 90089 USA; 3 Research Institute and Department of Gynecologic Oncology, Kanagawa Cancer Center, 2-3-2 Nakao, Asahi-ku, Yokohama, 241-8515 Japan; 4 Department of Internal Medicine, Division of Hematology-Oncology, Division of Gynecologic Oncology, University of Michigan, Ann Arbor, MI USA; 5 USC/Norris Comprehensive Cancer Center and Department of Obstetrics-Gynecology, Keck School of Medicine of USC, 1441 Eastlake Avenue, Los Angeles, CA 90089 USA; 6 Present Address: Magee-Womens Research Institute, University of Pittsburgh, Pittsburgh, PA USA; 7 Present Address: Genentech-Roche, 1 DNA Way, South San Francisco, CA USA

**Keywords:** Ovarian cancer, Prognostic markers

## Abstract

Epithelial ovarian cancer (EOC) is a leading cause of cancer-related mortality in the United States due to the late-stage disease at diagnosis. Overexpression of GRP78 and PDI following endoplasmic reticulum (ER) stress and activation of the unfolded protein response (UPR) promote growth and invasion in cancer. To identify novel prognostic biomarkers in EOC, here we determined the expression of ER stress-associated proteins (GRP78, ATF6 and PERK) and correlated with clinical outcome in EOC. Tissue microarray (TMA) samples from 415 tissues collected from three cancer centers (UM, USC, and KCCRI) were used to assess the expression levels of ER-associated proteins using immunohistochemistry (IHC). We observed that the expression levels of GRP78 (*p* < 0.0001), ATF6 (*p* < 0.0001), and PERK (*p* < 0.0001) were significantly increased in specimens of EOC compared to normal tissues, including in the serous subtype (*p* < 0.0001). Previously we reported that high expression of PDI correlated with poor patient survival in EOC. Here we showed that overexpression of GRP78 and PDI protein expression correlated with poor patient survival (*p* = 0.03), while low expression of combined GRP78 and PDI correlated with better survival (*p* = 0.01) in high-grade serous. The increased expression of ER stress-associated proteins in EOC suggests a role for ER stress and the UPR in EOC. More importantly, our results demonstrate that GRP78 and PDI are potential biomarkers for EOC and could be used as dual prognostic markers.

## Introduction

Epithelial ovarian cancers (EOC) are a leading cause of death from gynecologic malignancies and the fifth most common cause of cancer deaths in the United States, in large part because they are typically diagnosed at late stage^1^. Surgery and platinum/taxane-based chemotherapy form the mainstay of treatment^2^. Despite relatively high initial response rates, the majority of patients with advanced disease unfortunately recur following first-line treatment^2,3^. Previous studies showed that detection of the disease at an early stage is associated with prolonged survival, whereas patients diagnosed with advanced-stage EOC have significantly shorter survival rates^4–6^. Thus, early detection is still regarded as an important, albeit elusive, criterion for successfully treating and optimizing survival for patients with EOC. The commonly used biomarker, CA-125, to detect EOC is neither sensitive nor specific enough for early detection of the disease^4^. Hence, the discovery of new biomarkers is needed.

The activation of the unfolded protein response (UPR) and overexpression of chaperone proteins, 78 kDa glucose-regulated protein (GRP78) and protein disulfide isomerase (PDI), following ER stress promotes growth, survival, and invasion^7–13^. Cellular stress, due to the accumulation of misfolded/unfolded proteins, activates the UPR that halts the translation of proteins and stimulates the degradation of unfolded and aggregated proteins. Ultimately, the cell undergoes apoptosis unless protein homeostasis is restored^14^. Thus, in the presence of ER stress, cells either adapt and survive or undergo apoptotic/senescence cell death. Components of the UPR signaling pathway have potential clinical relevance in cancer because they influence tumor characteristics, are prognostic of clinical outcomes, and are therapeutically accessible^15,16^.

PDI is a redox chaperone upregulated during ER stress that serves as an important cellular defense against general protein misfolding^17^. Dysfunction of PDI in certain diseases causes additional ER stress due to the accumulation of unfolded proteins leading to initiation of the UPR. Triggering of UPR reduces the load of unfolded/misfolded proteins by induction of PDI and other chaperones through activation of ER sensors IRE1, PERK, and ATF6. GRP78 is a key regulator of UPR activation^18,19^.

Expression of ER stress markers such as GRP78 is increased in tissue specimens from endometrioid endometrial carcinomas at both mRNA and protein levels^20^. In addition, levels of GRP78 could affect the sensitivity to cisplatin by regulating autophagy and apoptosis in EOC^21–23^ and may be involved in EOC resistance to platinum-based therapy^24^. Like GRP78, PDI also has a dual role in cell death and survival during ER stress. Previously, we demonstrated that PDI is a potential biomarker for EOC^25^. GRP78 has also been reported as a potential independent predictor of recurrence and survival in prostate cancer^26^ and of responsiveness to chemotherapy in breast cancer^27^. Altogether, evidence suggests that targeting GRP78 and PDI could be an efficacious therapeutic option for EOC^28^.

In this study, we evaluated the expression of ER-associated proteins in EOC patient samples to identify predictive markers. We show that ER-associated proteins are highly expressed in EOC patient samples and that increased expression of GRP78 correlates with worse patient survival. Moreover, we demonstrate that low expression of combined GRP78 and PDI proteins correlate with better survival outcomes in EOC.

## Results

### Patient characteristics

Detailed patient characteristics are listed in Supplementary Table 1. Ovarian tumor tissue samples were collected from 415 patients who underwent treatment at USC, UM, and KCCRI. Paraffin-embedded tumor tissues from USC (89 cases), UM (192 cases) and KCCRI (134 cases) were used for the construction of the TMAs. The majority of patients were diagnosed with serous histology, late-stage, and/or high-grade disease. More than 50% of patients were diagnosed at the age of 55 or above (UM and KCCRI). The main clinical and pathological variables evaluated in this study are shown in Supplementary Table 1.

### Expression of ER proteins in ovarian cancer cell lines and xenografts

**Figure 1:**
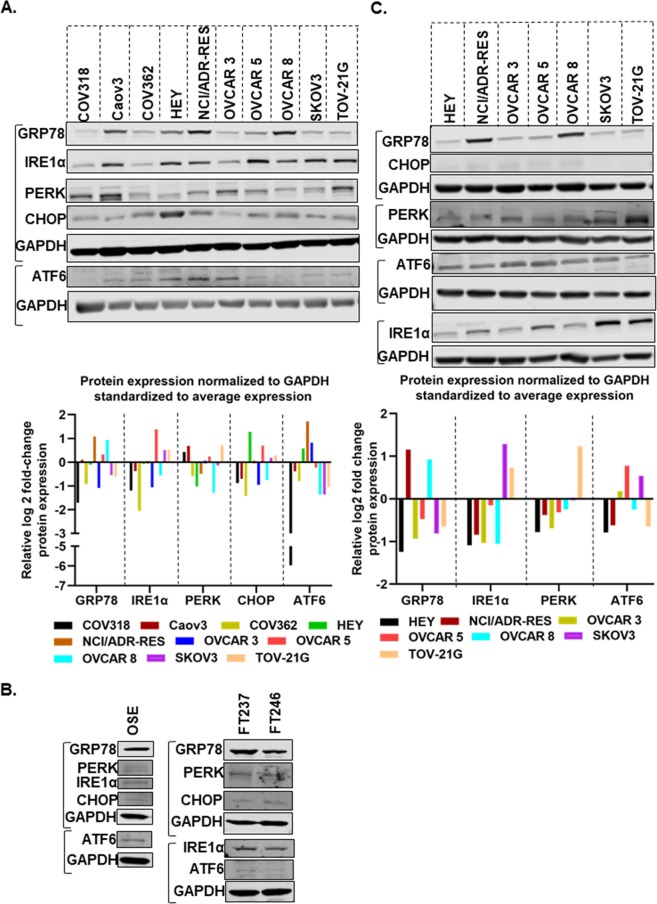
ER-stress associated proteins are highly expressed in ovarian cancer cells and xenograft models. (**A**) Protein expression levels of GRP78, ATF6, PERK, IRE1α, and CHOP in a panel of ovarian cancer cell lines. (**B**) Expression of ER-associated proteins in OSE (normal ovarian surface epithelial) and FT (normal fallopian tube) cells. (**C**) Expression levels of GRP78, ATF6, PERK, IRE1α, and CHOP proteins in a panel of ovarian cancer xenograft models. Relative band intensity of the protein of interest normalized to GAPDH using Image Studio software v5.2. Log 2 fold change of each protein expression standardized to the average expression in all cells was calculated and the relative log 2 fold change of protein expression is presented (GraphPad Prism 8.1.0). Each group of blots represents data obtained from a single gel and experiment. Different areas of the PVDF membrane are separated by white space.

Initially, we determined the expression of, GRP78, PERK, ATF6, IRE1α and CHOP in ten EOC cell lines by Western blotting (Fig. 1A and Supplementary Fig. S1A). Histopathology of the tumors from which the cell lines were derived is presented in Supplementary Table 2. It is important to note that the origin of some of those cell lines has been reassigned^29^, although there is controversy with these reassignments. GRP78 was expressed in almost all ten EOC cell lines. PERK and IRE1α had a moderate expression in the majority of cell lines. ATF6 expression was low in all cell lines. We also determined the expression of CHOP, a known ER stress marker that is usually upregulated following ER stress. CHOP expression was higher in HEY, NCI/ADR-RES and OVCAR 5 cell lines than other cells. To determine the expression signatures of each protein across the cell lines we used densitometric graphing of the log 2 fold change of each protein (normalized to the corresponding GAPDH) compared to the average expression in all cells (Fig. 1A lower panel). GRP78 was highly expressed in NCI/ADR-RES, OVCAR 5 and OVCAR 8 cells whereas in COV318, COV362, OVCAR 3, SKOV3 and TOV-21G cells GRP78 expression was lower than average expression. Expressions of IRE1α and CHOP were higher in OVCAR 5, SKOV 3 and TOV-21G however maximum fold change of CHOP expression was observed in HEY cell. In COV362, HEY, NCI/ADR-RES and OVCAR 8 cells PERK expression was lower than average. In a separate experiment, we determined the expression levels of ER-associated proteins in normal OSE (ovarian surface epithelial) cells, normal FT (fallopian tube) cells and other cancer cells as shown in Fig. 1B and Supplementary Fig. S1(B,C). Overall, expression of ER-proteins in normal OSE and FT cells was low, however GRP78 expression was higher among other ER proteins.

Next, we determined the expression of these proteins in eight EOC mouse xenografts (HEY, NCI/ADR-RES, OVCAR 3, OVCAR 5, OVCAR 8, SKOV3, and TOV-21G). The results are presented in Fig. 1C. Similar to protein expression in cell lines (Fig. 1A), GRP78 was highly expressed in tumor tissues collected from NIC/ADR-RES xenograft, IRE1α expression was higher in SKOV3 and TOV-21G xenograft tissues, and PERK expression was highest in TOV-21G xenograft compared to the average expression of each protein in all xenografts. However ATF6 expression was lower in the majority of the tissues compared to average expression and CHOP expression was low in all tissues.

### Immunohistochemical distribution of GRP78, PERK, and ATF6 in ovarian cancers

**Figure 2:**
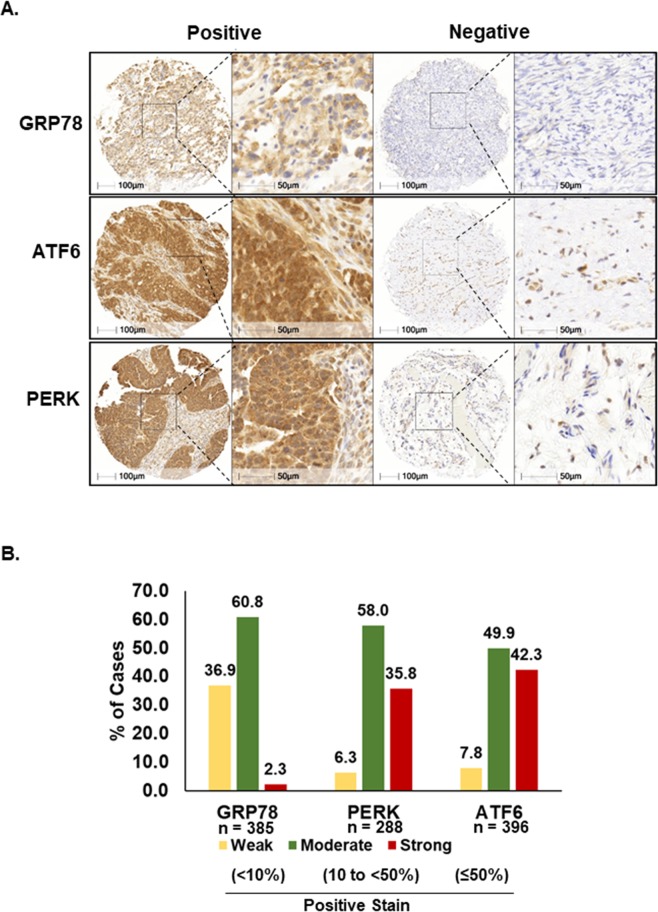
Immunohistochemical expression patterns of ER stress-associated proteins in ovarian cancer tissues. (**A**) Representative examples of positive and negative/weak staining patterns of GRP78, ATF6, and PERK in ovarian cancer tissues. (**B**) Percent of cases of strong (≥50%), moderate (10 to <50%), and weak (<10%) expression for each protein are shown. Percent of staining was automatically quantified by HALO (Indica labs, New Mexico, USA).

The expression of ER-associated proteins was evaluated in all TMAs. Digital image analysis (Halo, Indica labs) was used to determine the staining patterns. Of note, recent reports showed that digital image analysis accelerated the biomarker study to assess prognostic value with the perfect agreement to conventional assessment by expert pathologists^30,31^. Strong staining was defined as ≥50% of the tumor cells staining positive, moderate staining was defined as 10 to <50% of the tumor cells staining positive, and weak staining was defined as <10% of the tumor cells staining positive. Representative staining patterns (positive and negative/weakly stained) are depicted in Fig. 2A. Over half of the patients had ‘moderate’ expression for GRP78 and PERK and 49.9% showed ‘moderate’ expression of ATF6. Out of 385 ovarian carcinomas evaluated for GRP78 expression, 9 (2.3%) showed strong staining, 234 (60.8%) were moderate and 142 (36.9%) were weak (Fig. 2B). Among the 288 ovarian carcinomas stained for PERK, 103 (35.8%) demonstrated strong staining while 167 (58.0%) were moderate and 18 (6.3%) were weak. Finally, among 396 ovarian carcinomas evaluated for ATF6, 167 (42.3%) expressed strong levels, 197 (49.9%) moderate, and 31 (7.8%) showed weak staining.

### The expression of ER-associated proteins is correlated with patients’ pathological categories

**Figure 3:**
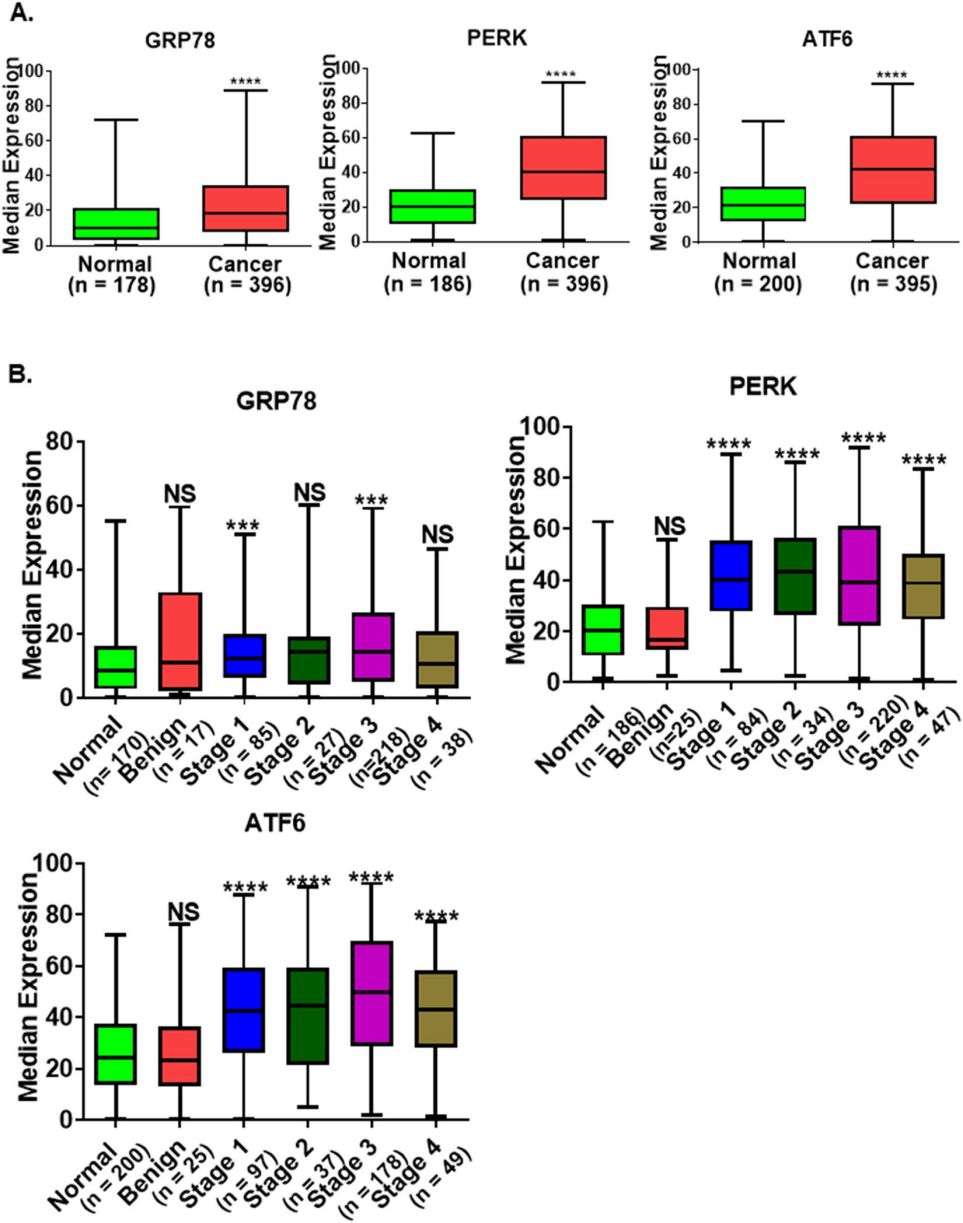
Expression of ER stress-associated proteins in ovarian cancer. (**A**) GRP78, PERK, and ATF6 are highly expressed in ovarian cancer compared to normal tissues. (**B**) Expression of ER stress-associated proteins in normal, benign and malignant tumor tissues collected from ovarian cancer patients with different stages, two US patient populations (USC and UM) and a Japanese patient population (KCCRI). *p*-values were calculated by the non-parametric Mann-Whitney U test in GraphPadPrism 8.1.0. ****p-value < 0.0001, ***p-value < 0.001, **p-value < 0.01, *p-value < 0.05, NS: not significant.

The majority of EOC patients had a moderate expression of GRP78, PERK, and ATF6. Next, we compared their protein expression in cancer cells to that of normal cells. ER proteins GRP78, PERK and ATF6 were highly expressed in EOC tissue compared to normal tissues: GRP78 (*p* < 0.0001), PERK (*p < *0.0001), ATF6 (*p* < 0.0001) (Fig. 3A). Since sample sizes were unequal we used non-parametric two-tailed Man-Whitney U test to confirm the statistical significance of the analyses. Representative staining of the ER stress proteins in non-tumor tissues is depicted in Supplementary Fig. S2.

We further investigated whether the expression of these proteins correlated with clinicopathological status. Expression of PERK and ATF6 significantly correlated with tumor stage (Stages I through IV: *p* < 0.0001); GRP78 expression also correlated with early tumor stages Stages I and III (*p* < 0.001) but not with Stage II and IV (Fig. 3B). The expression of GRP78, ATF6 and PERK were not different between benign tumors and normal tissues (Fig. 3C). Cumulatively, our data suggest that the expression level of ER proteins could be used as a diagnostic and prognostic biomarker.

### ER stress proteins are highly expressed in serous and clear cell ovarian carcinoma

**Figure 4:**
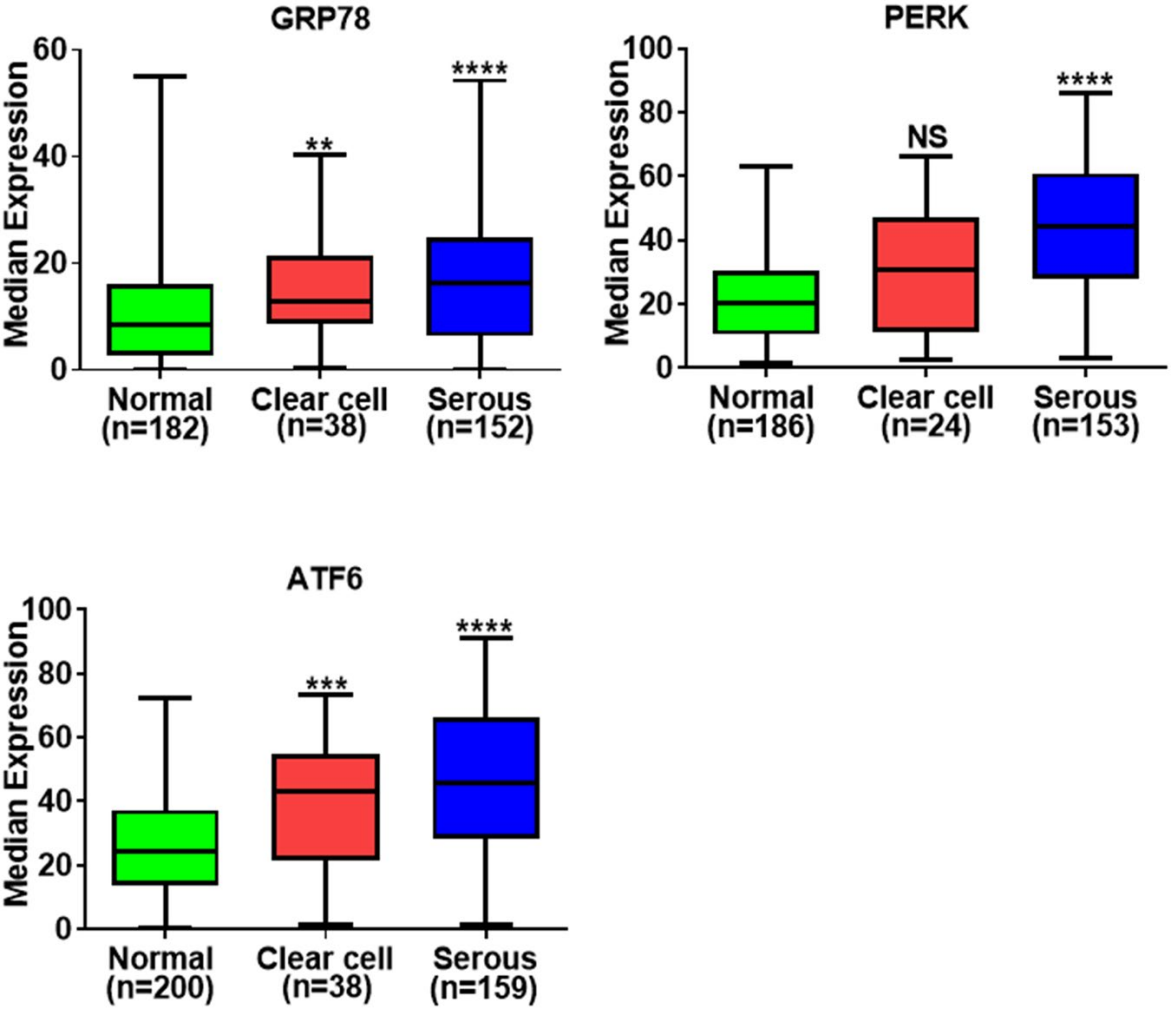
ER-associated proteins are overexpressed in serous and clear cell ovarian carcinoma. Protein expression levels of GRP78, PERK and ATF6 in clear cell and serous ovarian carcinoma. *p*-values were calculated by non parametric Mann-Whitney test in GraphPadPrism 8.1.0. ****p-value < 0.0001, ***p-value < 0.001, **p-value < 0.01, *p-value < 0.05, NS: not significant.

GRP78, PERK, and ATF6 were highly expressed in serous EOC (*p* < 0.0001) (Fig. 4). Examination of clear cell EOC tissues revealed that ATF6 and GRP78 were highly upregulated compared to normal tissues (*p* < 0.001 and *p* < 0.01) however PERK expression levels were not significantly increased (Fig. 4). We used the non-parametric Man-Whitney U test to determine the statistical significance of the analyses.

### Higher expression of GRP78 correlates with poor patient survival

**Figure 5:**
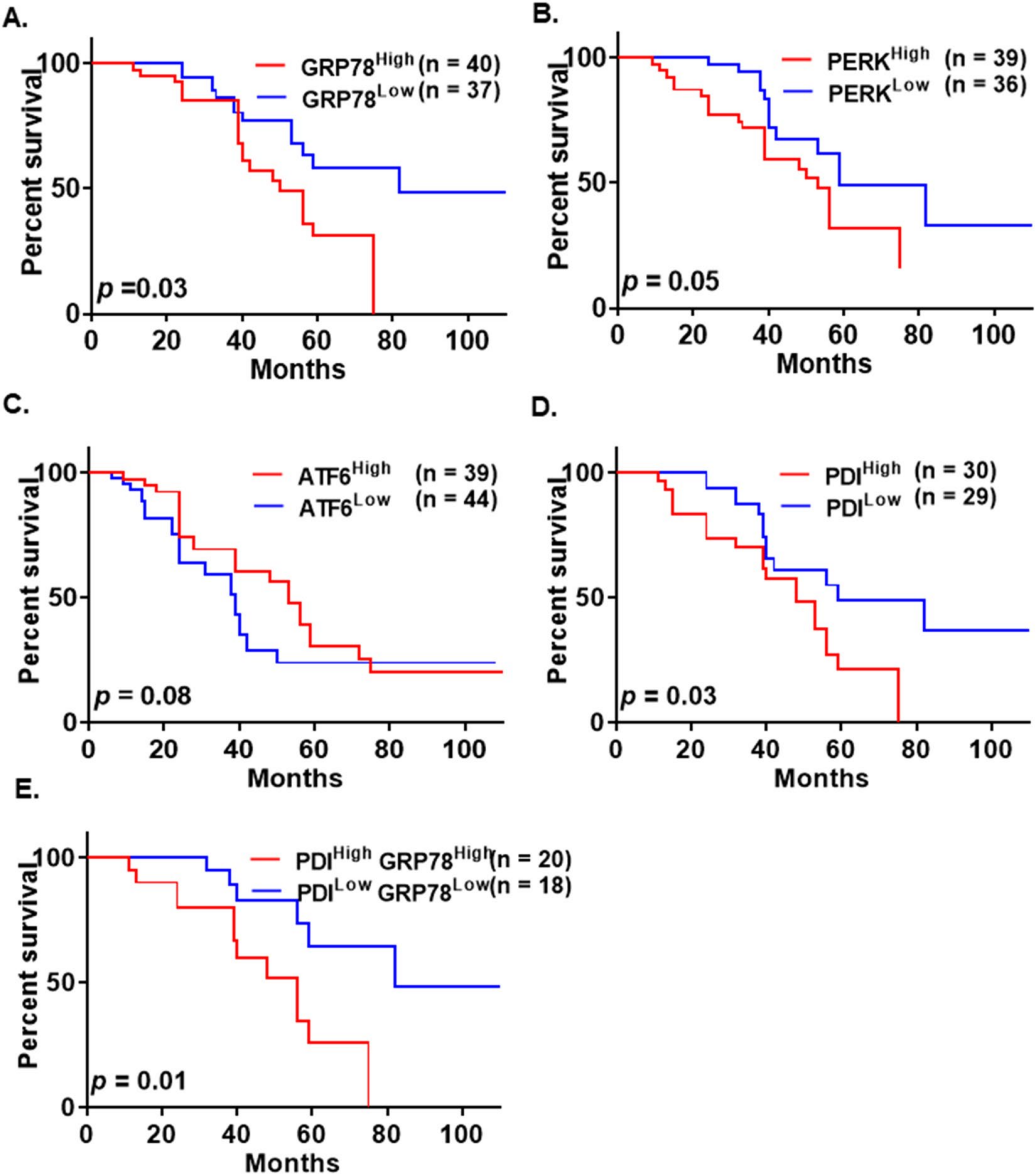
Correlation of GRP78, PERK ATF6 and PDI expression levels with overall survival rate in high-grade serous ovarian cancer patients. Overall survival curves for patients based on GRP78 (**A**), PERK (**B**), ATF6 (**C**), and PDI (**D**) expression levels. (**E**) Overall survival curve for EOC patients based on combined expression levels of GRP78 and PDI. Overall survival data sets were obtained from the UM subset of patients with high-grade serous EOC and analyzed with Prism 6 (GraphPad Software, Inc.). The Kaplan-Meier survival analysis method was used to generate survival curves using low (<median expression) and high expression (>median expression) groups. For the PDI survival curve, part of the data was used in previously published article^25^.

Kaplan-Meier analysis of overall survival was performed on the UM patient population to evaluate the prognostic significance of ER-related proteins PDI, GRP78, PERK, and ATF6 in high-grade serous type EOC. The high GRP78 expression group had a significantly worse overall survival than the low GRP78 expression group (*p = *0.03, Fig. 5A). There was no significant difference between high and low ATF6 and PERK expression with overall survival (Fig. 5B,C). We also observed that the high PDI expression group had a significantly worse overall survival than the low PDI expression group (*p* = 0.03, Fig. 5D). Kaplan–Meier analyses were also performed for overall survival based on combined GRP78 and PDI expression. Better separation between the overall survival curves of high-grade serous patients was observed for GRP78^high^PDI^high^ and GRP78^low^PDI^low^ expression (Fig. 5E) than the overall survival curves of EOC patients with single protein expression, GRP78^high^ and GRP78^low^ (Fig. 5A) or PDI^high^ and PDI^low^ expression (Fig. 5D). Therefore, we propose that the combined analysis of GRP78 and PDI expression can be clinically used as prognostic biomarkers in EOC.

## Discussion

Although GRP78 is well-known as a master regulator of ER stress, the clinicopathologic significance of GRP78 in EOC has not been well studied. In this study, we demonstrate that the expression of GRP78 is not only higher in EOC patient tissues compared to non-tumor tissues, but more importantly, it correlates with poor patient survival in high-grade serous type EOC. Previous studies have shown that overexpression of GRP78 expression was also identified as a prognostic factor in other cancers^32^.

Recently, we established that the high expression of PDI is a prognostic factor^25^ for EOC. GRP78 and PDI have functional similarities, for example, both increase folding capacity of protein in the ER to overcome stress, both are upregulated in several cancers and are independent prognostic factors for EOC. Our current study shows that high expression of both chaperone proteins, GRP78 and PDI, correlates with poor survival outcomes in high-grade serous EOC. This is perhaps due to the major roles that both proteins have in cancer progression. Thus, our finding suggests the potential value of applying these two biomarkers to clinical practice in EOC to determine disease aggressiveness.

Previous reports have shown that GRP78 and PDI overexpression in several cancers is due to the activation of UPR. UPR is considered to be a favorable mechanism for cancer progression, invasiveness, and resistance^33^. PERK, ATF6, and IRE1 are considered core components in UPR signaling and are important for cancer progression^34^. A recent report showed that activation of UPR signaling correlated to malignant progression and worse prognosis in prostate cancer^35^. In the present study, we determined that the expression of PERK and ATF6 was increased in EOC. The expression of these proteins was upregulated significantly with disease progression in EOC. Increased expression of ATF6 and PERK did not correlate with the overall survival of the high-grade serous patients. and further evaluation is needed to determine their prognostic value in EOC.

Due to the hypoxic condition and low nutrient levels of the tumor microenvironment, cancer cells are constantly experiencing activated ER stress and UPR. However, expression patterns of ER stress or UPR pathway proteins in clear cell and serous EOC were not previously studied. In our study, overexpression of GRP78, PERK, and ATF6 in EOC confirm that clear cell and serous EOC are under constant ER stress.

**Figure 6:**
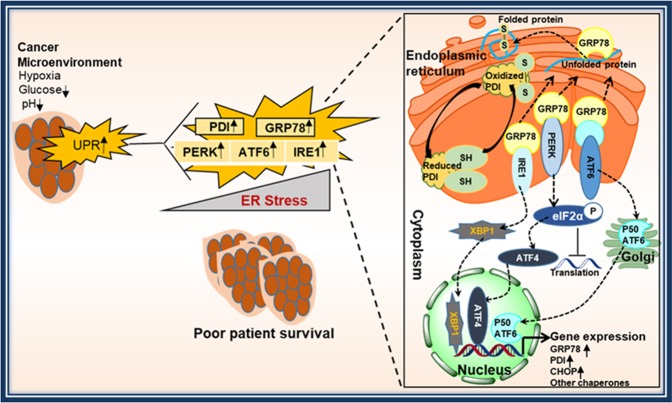
Schematic representation of UPR activation and patient outcome in cancer. The tumor microenvironment in EOC induces constant ER stress in cancer cells leading to the activation of the UPR pathway. The three branches of UPR: PERK, ATF6, and IRE1 have been implicated in cancer growth and progression. In several cancers, PERK, ATF6 and IRE1 expression along with the master regulators of ER stress, GRP78 and PDI are elevated compared to normal tissue. High levels of PDI and GRP78 can be used as biomarkers for the diagnosis and prognosis of EOC patients.

The ER-resident protein GRP78 binds to the luminal domain of all three UPR sensors (PERK, ATF6, and IRE1) and keeps these proteins in an inactive state under non-stress conditions. Upon ER stress, due to the accumulation of unfolded proteins, GRP78 dissociates from the UPR sensor proteins and activates a single branch or all three branches depending on the degree of stress. Activated PERK phosphorylates EIF2-alpha and inhibits global translation, but activates translation of ATF4 that increases expression of CHOP^34^. Both ATF4 and CHOP showed prognostic value in cancers. For example, Narita *et al*. reported low ATF4 expression correlated with shorter progression-free survival in multiple myeloma^36^. C/EBP homologous protein (CHOP) is reported as an independent prognostic marker for gastric cardia adenocarcinoma (GCA) patients. Low expression of CHOP correlated with poor prognosis of GCA patients^37^. Activation of IRE1 leads to XBP1 splicing, which stimulates the expression of UPR target genes^34^. A recent study showed that activation of the IRE1α-XBP1 pathway promoted cell proliferation and invasion by playing a major role in epithelial to mesenchymal transition in colorectal cancer. High expression of IRE1α also correlated with lower survival rates in colorectal cancer patients^38^. Overexpression of XBP1 showed a better outcome in multiple myeloma patients^39^. A recent meta-analysis demonstrated that low mRNA expression of XBP1 predicted poor prognosis in serous ovarian cancer^40^. In contrast, high XBP1 expression is associated with poor patient prognosis in glioma, triple-negative breast cancer and pre-B acute lymphoblastic leukemia (ALL)^41–43^. The third UPR branch, ATF6 signaling pathway, has been reported to play a role in disease recurrence and tumor growth. High expression of ATF6 is observed in recurrent tumors and correlated with poor prognosis in colon cancer^44,45^. During ER stress, ATF6 translocates to the Golgi apparatus and is cleaved by serine proteases to induce nuclear translocation of cleaved ATF6. In the nucleus, ATF6 binds to the ER stress response elements in the promoter region of GRP78 and induces transcription of GRP78. Similarly, components of IRE1 and PERK signaling pathways may also bind to the GRP78 promoter to elevate GRP78 transcription^46,47^. The roles of PDI in the regulation of ER stress sensor proteins are not well understood, however, PDI is an essential redox-sensitive activator of PERK^48^. In unstressed condition, ERp57 keeps PDI in a reduced state. In the absence of ERp57, PDI accumulates in the oxidized form that is necessary for the activation of PERK. All this evidence strongly supports that the UPR machinery has a prognostic value in cancer. However, the mechanisms of activation of UPR signaling factors in cancer growth or progression are poorly understood and need further investigation. Results from our study suggest that UPR activation elevates the expression of GRP78 and PDI, leading to poor survival of ovarian cancer patients. We suggest that activation of UPR factors may promote cancer progression. A schematic representation of the underlying mechanism is depicted in Fig. 6. We did not assess the changes in the expression of ER-proteins upon chemotherapy treatment due to incomplete patient information. However, a recent study on chemotherapy resistance in ovarian cancer showed that changes occurred in the gene expression profile after exposure to the neoadjuvant chemotherapy for high grade serous ovarian cancer patients^49^. From the gene list in that study, we found that FPKM (fragments per kilobase of exon per million fragments) levels of PDI decreased for all patients after treatment. FPKM levels of PERK is also decreased moderately in post-treatment. However, no changes observed for FPKM levels of ATF6 in five out of six patients. Given this information, it would be certainly interesting to determine the changes in the expression of ER–proteins in larger patient samples in the future.

In summary, ER-stress associated proteins GRP78, PERK, and ATF6 are highly expressed in clear cell and serous EOC patient samples compared to normal tissues, implying a role for ER stress pathways in EOC. Expression of these proteins increased significantly with tumor stages suggesting a role in EOC progression. More importantly, we demonstrated that GRP78 and PDI overexpression correlate with poor patient survival in high-grade serous type EOC. In conclusion, we propose that GRP78 and PDI together are independent prognostic factors for EOC. These dual biomarkers could be readily translated to the clinical practice to assess the aggressiveness of EOC at diagnosis and to monitor disease progression.

## Materials and Methods

### Patients and study population

Tissue specimens were collected from the Jean Richardson Gynecologic Tissue and Fluid Repository at USC (Los Angeles, CA), UM (Ann Arbor, MI), and KCCRI (Kanagawa Cancer Center Research Institute, Yokohama, Japan). Clinical information and follow-up data were obtained from medical records. All patients were staged according to the International Federation of Gynecology and Obstetrics (FIGO) classification and tumors were graded according to the World Health Organization (WHO) criteria.

### Ethics statement

Informed consent was obtained from all patients prior to tissue procurement. All studies were performed with the approval of the Institutional Review Boards of the University of Southern California and the University of Michigan, USA and Research Ethics Committee of Kanagawa Cancer Center Research Institute, Yokohama, Japan. We confirm that all research and methods were performed in accordance with the relevant guidelines and regulations.

### Normal controls

The term ‘normal’ used in this article was defined as normal ovarian tissue collected from (i) unaffected ovaries from patients at the time of removal of a paratubal cyst or follicular cyst (n = 2), (ii) normal ovarian tissue from the patient with ovarian cancer (n = 87) (UM). In the case of the Japanese samples, tissues were also collected from fallopian tubes, especially from the fimbriae (n = 134). During normal tissue collections, the corpus Albicans was excluded (KCCRI).

### Benign tumor

Benign tumor tissue collected from patients with benign adenofibromas (n = 2), serous cystadenomas (n = 11), mucinous cystadenomas (n = 6), fibroid (n = 1), struma ovarii (n = 1), and mature teratoma (n = 1).

### Tissue microarray (TMA)

Formalin-fixed and paraffin-embedded primary tumor and non-tumor tissues from EOC patient samples were used for TMA construction in this study. After review and confirmation by the histopathologist from each center, TMAs were constructed as described by Kononen *et al*.^50^. Briefly, after carefully choosing the morphologically representative region from the hematoxylin-eosin section, either 1 mm, 2 mm, or 4 µm cores (for samples collected from USC, KCCRI, or UM, respectively) were punched from the selected paraffin-embedded donor blocks and transferred to the paraffin-embedded receiver block. To overcome tumor heterogeneity, core biopsies were performed from two or three different areas of each tumor, and tissues were spotted in duplicate or triplicate on the TMA slides.

### Immunohistochemistry and automated analysis of immunohistochemistry

Immunohistochemical (IHC) staining was performed by the Pathology Core at the University of Michigan as previously described^25^. Briefly, IHC staining was used to assess protein expression on TMA slides. Following antigen retrieval with Diva, quenching of endogenous peroxidase, and rodent block treatments (Biocare), slides were incubated with primary rabbit antibodies [PDI/P4HB (11241-1-AP), GRP78/HSPA5 (11587-1-AP), PERK/EIF2AK3 (24390-1-AP), and ATF6 (24169-1-AP)] from Proteintech] for 30–60 minutes. After primary antibody incubation and washing, rabbit polyclonal HRP secondary antibody (Biocare) was applied. Negative controls were obtained by substitution of the primary antibody with Universal Negative reagent (Biocare). Following washing, 3,3-diaminobenzidine (DAB) was applied to visualize all reactions, and slides were counterstained with hematoxylin. The sections were dehydrated through graded alcohols, immersed in xylene, and mounted with coverslips. The staining in tumor areas was confirmed by a pathologist. We omitted the tissue spots in the TMA slide with poor staining that caused the variation of the numbers in the different cohorts and histology subtypes (e.g cancer/normal/benign) for each protein biomarker staining.

TMA slides were scanned using a high throughput pannoramic scanner. Images were visualized by the Case viewer and the percentage of positive staining was calculated by HALO (Indica Labs) software package. Positivity was quantified as the number of positive pixels/mm^2^. Strong staining was considered as ≥50% positively stained tissue, moderate staining was defined as 10 to <50% positively stained tissue, and weak staining was defined as <10% positively stained tissue.

### Animal models

All animal experiments strictly followed the guidelines of the Animal Ethics Committee of the University of Michigan. All animal studies were approved by the Institutional Animal Care & Use Committee (IACUC) of the University of Michigan (PRO00009185). Five-Six week old female nude mice obtained from Taconic (USA) used for the experiment. We confirm that all research and methods were performed in accordance with the relevant guidelines and regulations.

### Development of mouse xenograft

Ten EOC cell lines (COV318, Caov3, COV362, HEY, NCI/ADR-RES, OVCAR 3, OVCAR 5, OVCAR 8, SKOV3, and TOV-21G) were cultured (see Supplementary Section for detailed cell culture and xenograft method) and approximately 2–4 × 10^6^ cells were injected subcutaneously into each mouse to generate xenografts. When tumor size reached approximately 1,000 mm^3^ animals were euthanized and tissue samples were collected for IHC staining and Western blotting.

### Preparation of tumor lysate and western blotting

Western blot samples were prepared as described^25^. Briefly, tissue samples stored at −80 °C were thawed in RIPA buffer (200 μL to 400 μL) supplemented with proteinase- and phosphatase-inhibitor cocktail (Sigma), then homogenized with an electrical homogenizer followed by short sonication to form a homogeneous tissue lysate. The lysate solution was centrifuged at 18,000 × g for 30 min at 4 °C. Protein concentration was measured with the BCA assay (Thermo Fisher). Cell lysate of fallopian tube cells FT237 and FT246 were obtained from Dr. Analisa Difeo (University of Michigan, MI). Thirty to forty μg protein per sample was subjected to SDS-PAGE analysis. The lysates of ovarian surface epithelial cells and fallopian tube cell 15–20 µg protein was used for WB analysis. Proteins were then electro-transferred to methanol activated immobilon-FL PVDF membranes (EMD Millipore). Membranes were blocked with Starting Block (Thermo Fisher) for 1 hr at room temperature and incubated with primary antibodies overnight at 4 °C. Dylight 800-conjugated secondary antibodies were used for detection (Thermo Fisher, 1:5000, 5% milk, 1 hour, RT) of fluorescent signals in Odyssey Imaging Systems (LI-COR Biosciences).

### Antibodies used for western blot

The antibodies used for Western blot are, GRP78/HSPA5 (11587-1-AP), PERK/EIF2AK3 (24390-1-AP), and ATF6 (24169-1-AP) from Proteintech, Rosemont, IL, USA. IRE1α (3294) and CHOP (2895) antibodies were purchased from Cell Signaling Technology (Danvers, MA, USA).

### Statistical analysis

Statistical analysis was evaluated using the non-parametric Mann-Whitney U test (GraphPad Prism 8.1.0). Two-tailed *p*-value < 0.05 was considered statistically significant. Survival curves were generated using overall survival (OS) data from the UM cohort. For survival curves, we used the conventional Kaplan-Meier method and compared curves using the log-rank test and *p*-values < 0.05 were considered to be statistically significant.

## Supplementary information

Supplementary information.

## Data Availability

The datasets generated for the current study are available from the corresponding author upon request.
