# Supplementary material for: Clinicopathological significance of endoplasmic reticulum stress proteins in ovarian carcinoma
Source: Sci Rep. 2020 Feb 7;10:2160. doi: 10.1038/s41598-020-59116-x (PMC7005787; doi:10.1038/s41598-020-59116-x)
Supplement: Supplementary file 1 — Supplementary information. [file 41598_2020_59116_MOESM1_ESM.pdf]

Supplementary information

Clinicopathological significance of endoplasmic reticulum stress proteins in ovarian carcinoma

Soma Samanta[1], Shuzo Tamura[1], Louis Dubeau[2], Paulette Mhawech-Fauceglia[2], Yohei Miyagi[3], Hisamori Kato[3], Rich Lieberman[4], Ronald J. Buckanovich[4, a], Yvonne G. Lin[2, b] and Nouri Neamati[1]

[1]Department of Medicinal Chemistry, College of Pharmacy, Rogel Cancer Center, University of Michigan, 1600 Huron Parkway, Ann Arbor, MI 48109, USA

[2]USC/Norris Comprehensive Cancer Center and Department of Pathology, Keck School of Medicine of USC, 1441 Eastlake Avenue, Los Angeles, CA 90089, USA

[3]Kanagawa Cancer Center Research Institute, 2-3-2 Nakao, Asahi-ku, Yokohama 241-8515, JAPAN

[4]Department of Internal Medicine, Division of Hematology-Oncology, Division of Gynecologic Oncology, University of Michigan, Ann Arbor, MI, USA

[a]Current address: Magee-Womens Research Institute, University of Pittsburgh, Pittsburgh, PA.
[b]Current address: Genentech-Roche, 1 DNA Way, South San Francisco, CA.

Correspondence and requests for materials should be addressed to N.N. (email: neamati@umich.edu)

Methods

Cell Culture

OVCAR 8, OVCAR 5, and OVCAR 3 cells (National Cancer Institute, Developmental Therapeutics Program, Bethesda, MD) were maintained in RPMI-1640 supplemented with 10% heat-inactivated FBS (Gemini-Bioproducts). NCI/ADR-RES cells (National Cancer Institute) were maintained in RPMI-1640 supplemented with 10% heat-inactivated FBS and 5 mmol/L L-glutamine. HEY and Caov-3 cell lines were kindly provided as a gift by Dr. Louis Dubeau (University of Southern California, Keck School of Medicine, Los Angeles, CA). HEY cells were maintained in Dulbecco's Modified Eagle Medium (DMEM) supplemented with 10% heat-inactivated FBS and 5 mmol/L L-glutamine. Caov3 cells were maintained in MEM supplemented with 10% heat-inactivated FBS. Cells were grown as monolayers at 37ºC in a humidified atmosphere of 5% CO_2. COV318, COV362, SKOV3 and TOV-21G cells (National Institute of Health, Bethesda, MD) were maintained in DMEM (COV318 and COV362) and RPMI (SKOV3 and TOV-21G) with 10 % FBS. To remove adherent cells from the flask for subculture and counting, cells were washed with PBS without calcium or magnesium, incubated with a small volume of 0.25% trypsin–EDTA solution (Mediatech, Inc.) for 5 to 10 minutes, re-suspended with culture medium, and centrifuged. All experiments were carried out using cells in the exponential growth phase. All cells were authenticated by short tandem repeat (STR) at the University of Michigan Genomics Core facility. Cells were routinely checked for mycoplasma contamination by using PlasmoTest (InvivoGen).

Non-tumor control

The term 'normal' used in this article was defined as non-tumor tissues collected from normal ovaries from patients at the time of removal of paratubal or follicular cysts and from

patients with ovarian cancer (UM). Because of the recent consensus on the origin of the EOC, non-tumor tissues were also collected from fallopian tubes, especially from the fimbriae. During normal tissue collections, the corpus albicans was excluded (KCCRI). Representative stainings of the ER stress proteins in non-tumor tissues are depicted in Supplementary Figure S2. We pooled all the normal tissues collected from UM and KCCRI and compared the expression of ER stress-associated proteins GRP78, PERK and ATF6 against tumor tissues collected from all three patient populations (USC, UM and KCCRI). Two hundred and twenty-three non-tumor –control tissues were used in this study.

Development of mouse xenograft

Ten EOC cell lines (COV318, Caov3, COV362, HEY, NCI/ADR-RES, OVCAR 3, OVCAR 5, OVCAR 8, SKOV3, and TOV-21G) were cultured as described above. Histopathology of the tumors from which the cell lines were derived are sourced from our previous report[18] and presented in Supplementary Table 2. Approximately 2-4 $x10^6$ cells of a single cell line were injected subcutaneously into each mouse. We generated xenografts from 7 cell lines (HEY, NCI/ADR-RES, OVCAR 3, OVCAR 5, OVCAR 8, SKOV3, and TOV-21G). When tumor size reached approximately 1,000 mm^3, animals were euthanized and tissue samples were collected. Half of the mass was preserved for IHC staining and the other half was flash-frozen in liquid nitrogen and stored at -80 ˚C for Western blotting.

Supplementary Table 1. Clinico-pathological characteristics of the patients sample

**Patient population**	**USC**		**UM**		**KCCRI**	
**Patients' number**	89		192		134	
**Age (year)**						
**≥55**	26 (29.2%)		130 (67.7%)		84 (62.7%)	
**<55**	44 (49.4%)		62 (32.3%)		50 (37.3%)	
**Not known**	19 (21.3%)		-		-	
**Histology**	Serous	35 (39.3%)	Serous	152 (79.2%)	Serous	51 (38.1%)
	Endometroid	17 (19.1%)	Endometroid	1 (0.5%)	Endometroid	18 (13.4%)
	Clear Cell	10 (11.2%)	Clear Cell	3 (1.6%)	Clear Cell	38 (28.4%)
	Mucinous	17 (19.1%)	Mucinous	0 (0%)	Mucinous	15 (11.2%)
	Others	10 (5.6%)	Other	36 (18.7%)	Other	11 (8.2%)
**Type**	**Benign**	8 (9.0%)	**Benign**	12 (6.3%)	**Benign**	1 (0.7%)
	**FIGO Stage**					
	I	36 (44.4%)	I	9 (4.8%)	I	56 (41.8%)
	II	6 (7.4%)	II	17 (9%)	II	16 (11.9%)
	III	31 (38.8%)	III	112 (59.3%)	III	40 (29.9%)
	IV	8 (9.8%)	IV	28 (14.8%)	IV	16 (11.9%)
			Other	14 (7.2%)	Others	5 (3.7%)
	**Grade**					
	I	19 (28.3%)	I	19 (9.9%)		
	II	6 (8.9%)	II	4 (2.1%)	NA	
	III	42 (62.7%)	III	134 (69.8%)		
	Others	14 (15.7%)	Others	23 (12.0%)		
**Type of Chemotherapy**	No chemotherapy	31 (34.8%)	NA		No chemotherapy	6 (4.5%)
	Platinum containing	5 (5.6%)			Platinum containing	3 (2.2%)
	Platinum and taxol containing	53 (59.5%)			Platinum and taxol containing	107(79.9%)
					No Information	18 (13.4%)
**Race**	Asian	20 (22.5%)	NA			
	Black	2 (2.2%)				
	Native American	3 (3.3%)				
	White	64(71.9%)				

Supplementary Table 2

Histology of the ovarian cancer cell

Cell lines	Histology
Caov3	Serous[1]/High grade serous[2]
COV318	Serous/ High grade serous[3]
COV362	Endometroid[1]/ Likely High grade serous[2,3]
HEY	High grade serous[2]
NCI/ADR-RES	High grade serous[4]
OVCAR 3	High grade serous[3]
OVCAR 5	High grade serous[3]
OVCAR 8	High grade serous[3]
SKOV3	Serous[5]/Atypical non serous cell lines[2]/unlikely high grade serous[6]/clear cell carcinoma[3]
TOV-21G	Clear cell carcinoma[2]

Supplementary references

1 Clarke, H. J., Chambers, J. E., Liniker, E. & Marciniak, S. J. Endoplasmic reticulum stress in malignancy. *Cancer Cell* **25**, 563-573, doi:10.1016/j.ccr.2014.03.015 (2014).

2 Anglesio, M. S. *et al.* Type-specific cell line models for type-specific ovarian cancer research. *PLoS One* **8**, e72162, doi:10.1371/journal.pone.0072162 (2013).

3 Beaufort, C. M. *et al.* Ovarian cancer cell line panel (OCCP): clinical importance of in vitro morphological subtypes. *PLoS One* **9**, e103988, doi:10.1371/journal.pone.0103988 (2014).

4 https://web.expasy.org/cellosaurus/CVCL_1452.

5 Hernandez, L. *et al.* Characterization of ovarian cancer cell lines as in vivo models for preclinical studies. *Gynecol Oncol* **142**, 332-340, doi:10.1016/j.ygyno.2016.05.028 (2016).

6 Domcke, S., Sinha, R., Levine, D. A., Sander, C. & Schultz, N. Evaluating cell lines as tumour models by comparison of genomic profiles. *Nat Commun* **4**, 2126, doi:10.1038/ncomms3126 (2013).

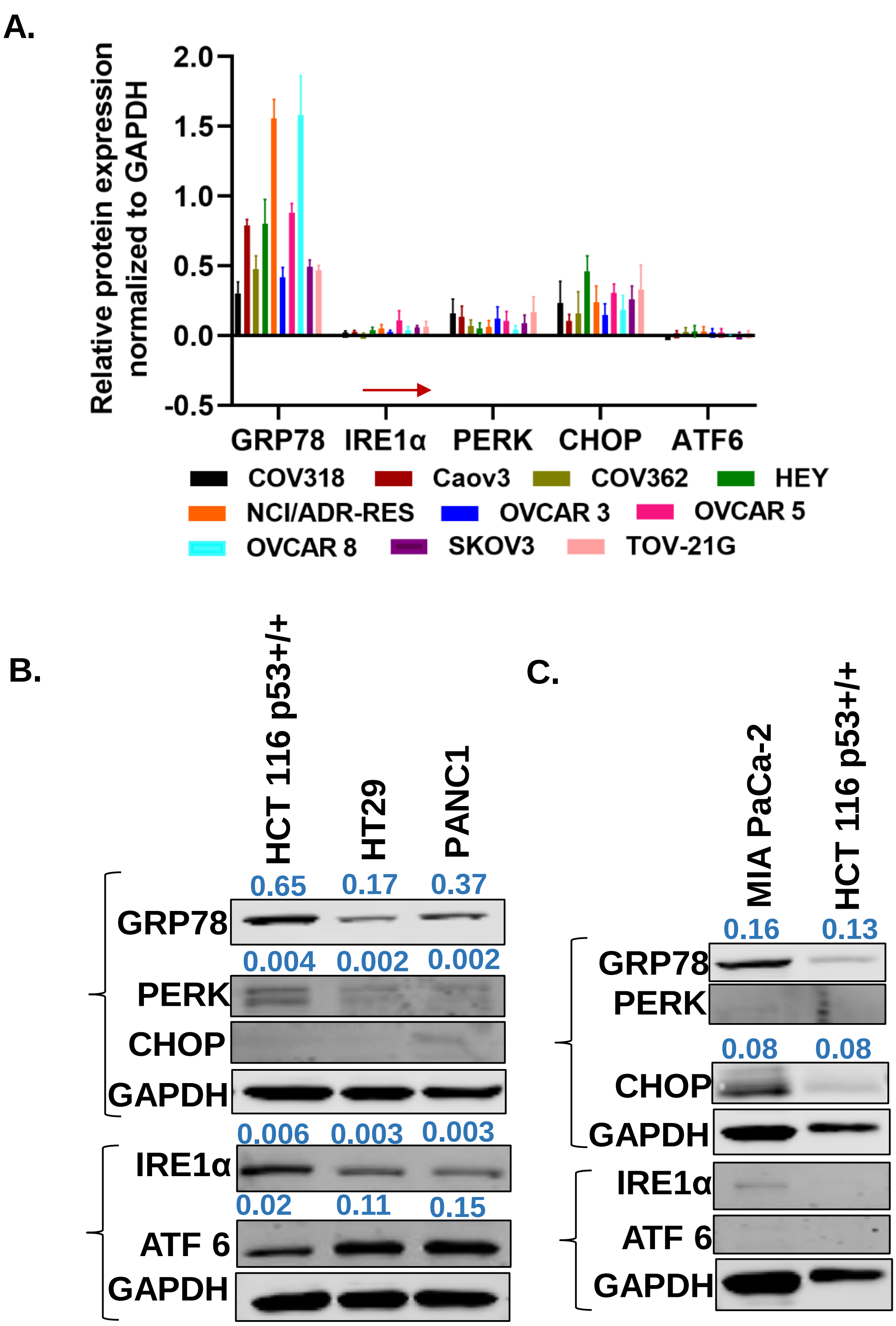
A.
Relative protein expression normalized to GAPDH
2.0
1.5
1.0
0.5
0.0
-0.5
GRP78
IRE1α
PERK
CHOP
ATF6
COV318
Caov3
COV362
HEY
NCI/ADR-RES
OVCAR 3
OVCAR 5
OVCAR 8
SKOV3
TOV-21G
B.
HCT 116 p53+/+
HT29
PANC1
0.65
0.17
0.37
GRP78
0.004
0.002
0.002
PERK
CHOP
GAPDH
0.006
0.003
0.003
IRE1α
0.02
0.11
0.15
ATF 6
GAPDH
C.
MIA PaCa-2
HCT 116 p53+/+
0.16
0.13
GRP78
PERK
0.08
0.08
CHOP
GAPDH
IRE1α
ATF 6
GAPDH

Supplementary Fig. S1. ER-stress associated proteins are highly expressed in ovarian cancer, other cancer cells, and xenograft tissues. (A) Protein expression levels of GRP78, PERK, IRE1α, CHOP, and ATF6, in ovarian cancer cell lines. Relative protein expression normalized to GAPDH is plotted in GraphPad Prism 8.1.0. Expression of each proteins from three independent experiments are presented as mean ± SD. (B) Protein expression levels of GRP78, ATF6, PERK, IRE1α, and CHOP in representative colon and pancreatic cancer cell lines. (C) Expression of ER-associated proteins in representative colon and pancreatic cancer xenograft tissues. Relative band intensity of the protein of interest normalized to GAPDH using Image Studio software (version 5.2). Each group of blots represents data obtained from a single gel and experiment. Different areas of the PVDF membrane are separated by white space.

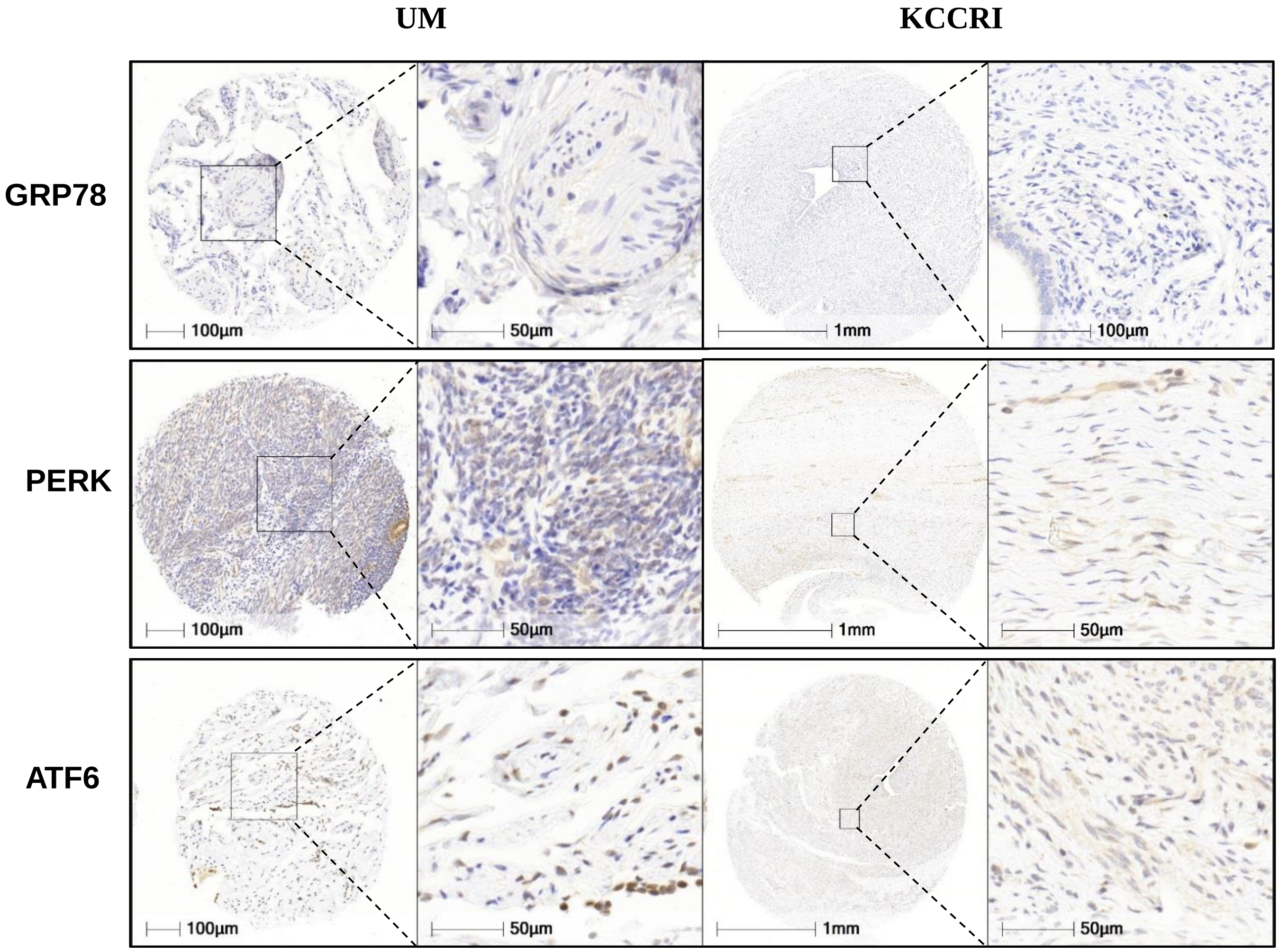

Supplementary Fig. S2. Representative staining of ER-stress associated proteins in non-tumor control sample from UM (USA) and KCCRI (Japan) cohort.

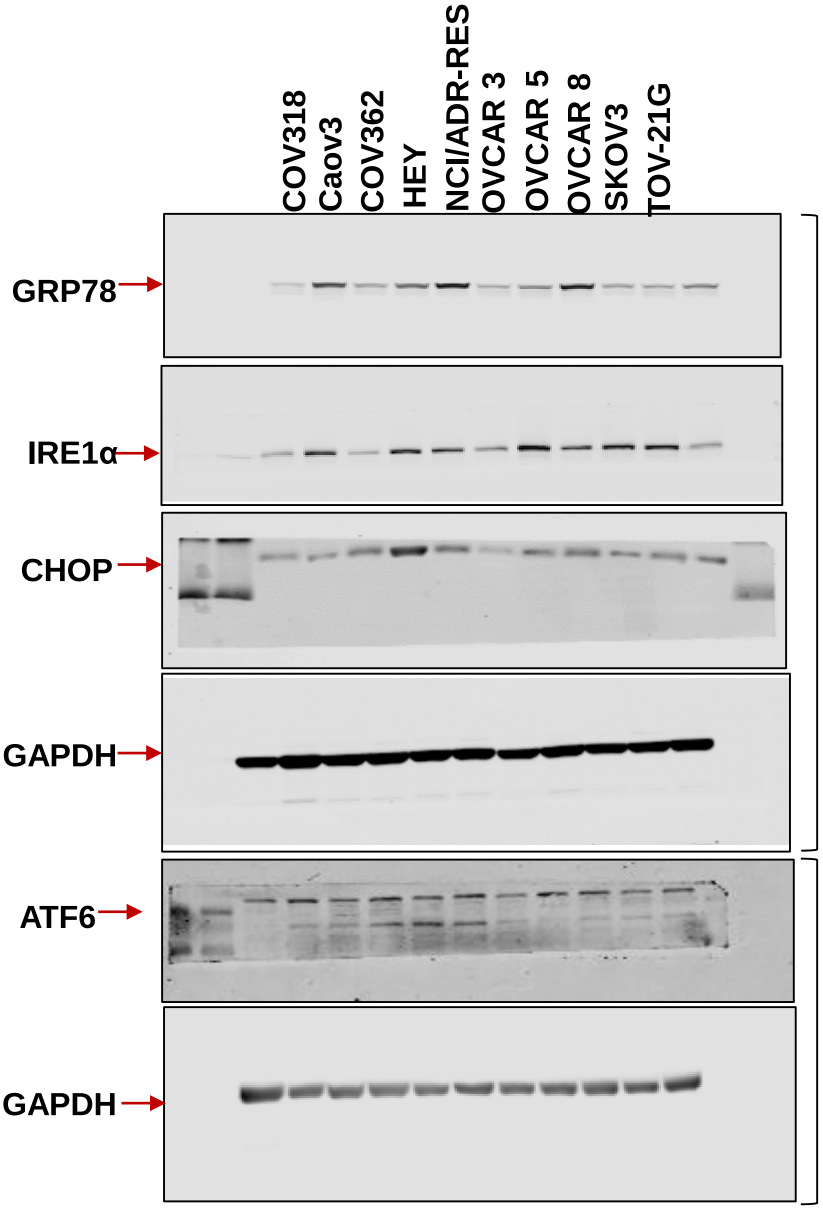

Supplementary Fig. S3. Figure 1A un-crop blots, for each protein. Each group of blots represents data obtained from a single gel and experiment. Different areas of the PVDF membrane are separated by white space.

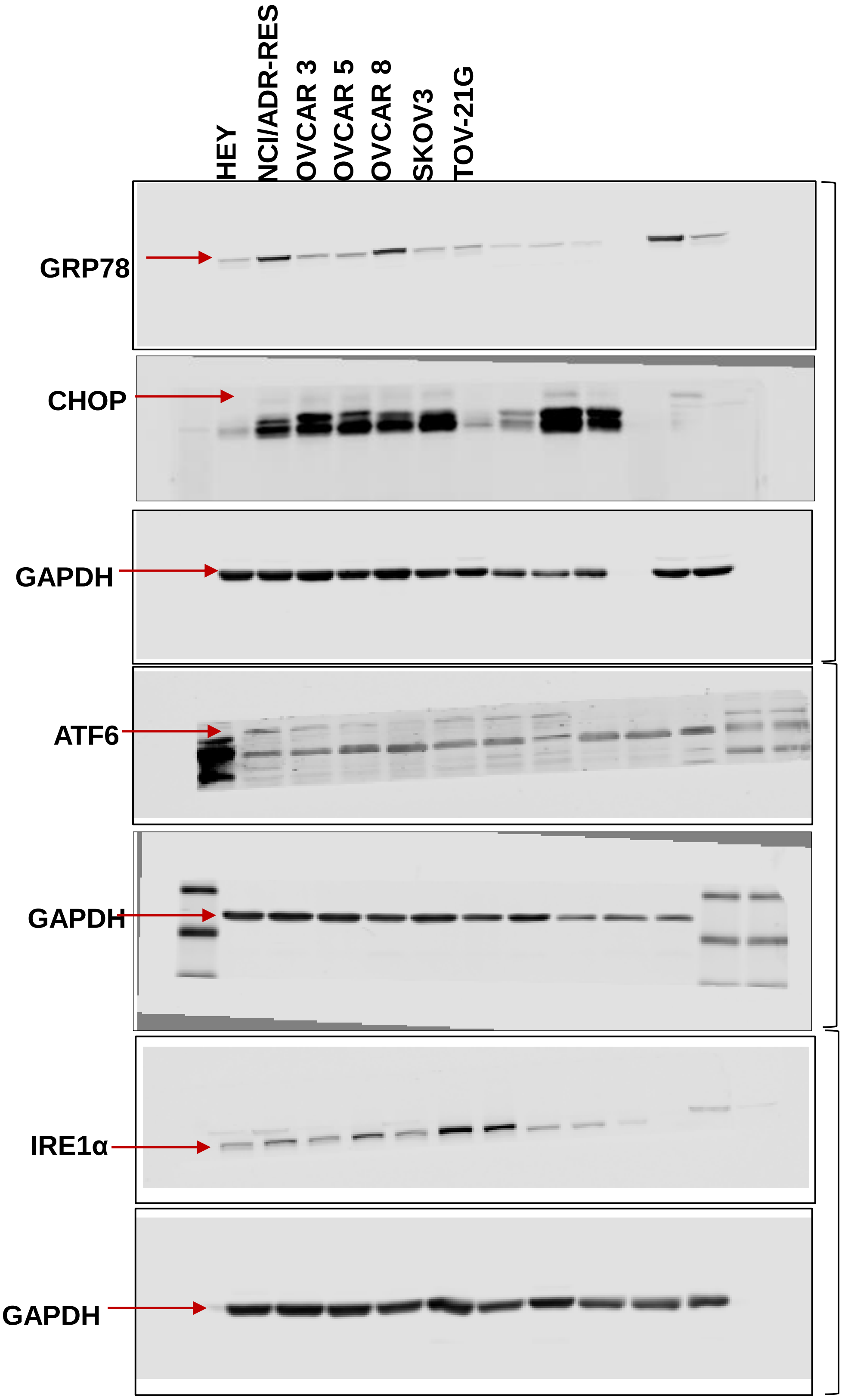

Supplementary Fig. S4. Figure 1C un-crop blots, for each protein. Each group of blots represents data obtained from a single gel and experiment. Different areas of the PVDF membrane are separated by white space.

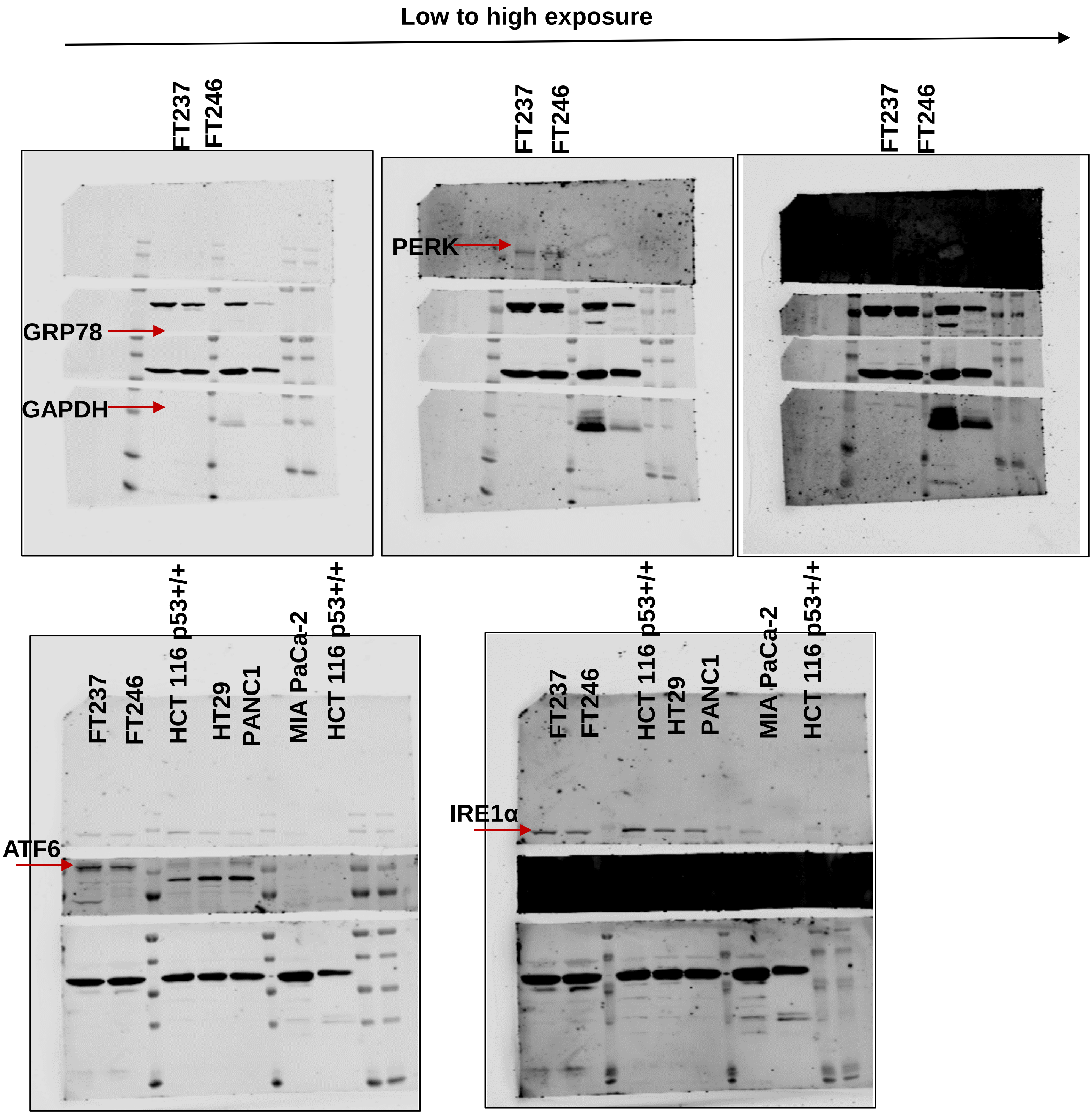

Supplementary Fig. S5. Figure 1B, supplementary figure 1B and 1C un-crop blots, with the different exposure times used for each protein. Each group of blots represents data obtained from a single gel and experiment. Different areas of the PVDF membrane are separated by white space.

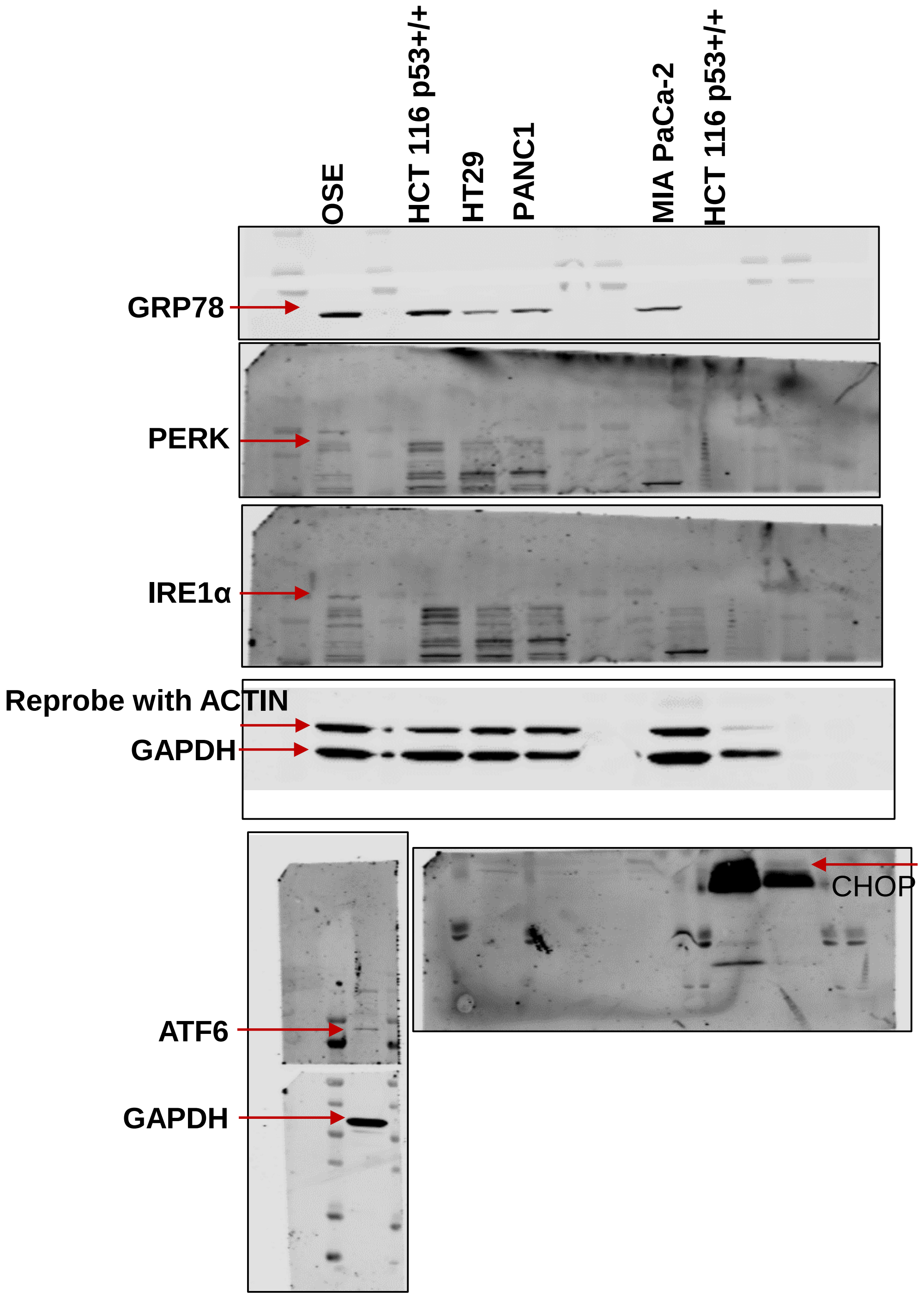

Supplementary Fig. S6. Figure 1B, supplementary figure 1B and 1C un-crop blots, with the different exposure times used for each protein. Each group of blots represents data obtained from a single gel and experiment. Different areas of the PVDF membrane are separated by white space.
